# Process Changes for Stroke Care and Electroencephalography on a Neurology Service in a Hospital at the Epicenter of the COVID-19 Pandemic

**DOI:** 10.3389/fneur.2020.605315

**Published:** 2021-01-08

**Authors:** Helen Valsamis, Noa Sheikin, Susan Law, Samah Abdul Baki

**Affiliations:** ^1^Kings County Hospital Center, SUNY Downstate Medical Center, Brooklyn, NY, United States; ^2^Bio Signal Group, Brooklyn, NY, United States

**Keywords:** change in process, EEG, critical care, stroke, COVID-19

## Summary

The United States, with over 11 million cases and ~250,000 deaths (1), has become the epicenter of the COVID-19 worldwide pandemic since the first case was identified in Washington State on January 19, 2020.

In New York City the first case of community acquired COVID-19 was identified on March 1, 2020 and the number of known cases increased rapidly making the city the epicenter of COVID-19 in the United States. Public hospitals became deluged with patients as the communities they serve, urban poor and minority, were disproportionately affected by the disease. COVID-19 often affects the nervous system with both central and peripheral sequalae, neurology services had to adapt to a new landscape (2–6).

This paper will report on the process changes for the neurology service, in particular for stroke and electroencephalography (EEG) services, at King County Hospital Center (KCHC), a 637 bed, public, university-affiliated, teaching hospital in central Brooklyn, New York, which serves a predominantly African American community. Implementing those changes resulted in maintaining our pre-COVID structure and quality of care despite the workflow, economic, and technical challenges induced by the virus.

## KCHC as COVID Surges in New York City

The first known COVID-19 patient was admitted to KCHC on March 13, 2020, almost 2 weeks after the first known case of COVID-19 was identified in New York City. The number of cases rapidly increased first in Manhattan and then in Brooklyn before in Queens and then in the Bronx. In March and April as case numbers surged at KCHC, strategies to protect patients and staff were developed and implemented rapidly as we learned first-hand about the disease.

## Neurology Ppe Policy Within the Hospital-Wide Response

Initially, COVID-19 was thought to be a respiratory disease. However, in the first few days of the surge of patients, multiple Neurology and Emergency Medicine personnel were exposed to COVID-19 patients who presented with strokes but without respiratory complaints. The Neurology Service responded by promptly instituting a policy that full personal protective equipment (PPE) including N95 masks and face shields be used for performing stroke codes, adapting procedures for performing neurologic examinations, and changing the workflow for EEG. We also reorganized our services and participated in many facets of the hospital-wide COVID-19 response

## Staffing and Service Restructuring

As was the case in many other hospitals when COVID-19 arrived, our Neurology Department made staffing changes to support the COVID-19 effort. We disbanded our Neuro Critical Care Service and sent the attendings into the Intensive Care Unit (ICU) attending pool as the hospital expanded its ICU bed capacity from 40 to 200 ICU beds. The Neurology Consultation Service provided neurology guidance on critically ill patients with neurologic conditions. Our Neuro Critical Care attendings graciously answered our questions on difficult to manage patients. Our current stroke fellow is also an Emergency Medicine attending and he put his fellowship on hold and went full time to the Emergency Department (ED) during the peak of the crisis.

The inpatient Neurology Service expanded to become a combined Neurology-COVID service caring for patients with neurological conditions, COVID-19, or both. We received training on the evolving management of COVID-19 from Infectious Disease and ICU attendings. We also held a journal club on the neurologic manifestations of COVID-19 and regularly emailed pertinent articles to the entire Neurology faculty and all trainees. The Pulmonary Service consulted regularly on our patients on mechanical ventilation and BIPAP. The infectious disease attendings came by on a daily basis to advise us on both the management of COVID-19 and multiple other medical issues. Our Adult Neurology residents and attendings also rotated onto the Medicine services which were almost all COVID-19 wards. Of the two approaches, expanding the scope of the Neurology-COVID-Medicine service worked better. We maintained our pre-COVID structure and were better able to maintain the morale of our teams.

On the outpatient side, we rapidly transitioned from in-person visits to telephone visits (televisits) and eventually our Stroke Clinic instituted video visits. Electromyography and outpatient EEG studies were suspended until the surge passed. Our Pediatric Neurology fellows performed nasopharyngeal swabs for outpatient and employee testing. Our Adult and Pediatric Neurology attendings helped out Employee Health with the phone calls to quarantined staff. Our Pediatric Neurology fellows also rounded with the ICU teams and served as the liaison with the families who were not allowed to visit their loved ones.

Our Stroke Nurse Practitioner became the PPE trainer for stroke codes but also for trauma codes. She quickly trained all of the staff on both Trauma and Neurology in proper PPE donning, use, and doffing. In addition, she developed Stroke code kits comprised of N-95 masks, face shields, gowns, bonnets, and gloves, so the responder had a pre-assembled set of PPE and could rapidly prepare to safely answer a stroke code.

## Stroke Codes, Neurological Exam, Aphasia Testing, and Pupil Examinations

We also analyzed and adapted the workflow of the stroke code and neuro exam. Prior to the pandemic, we used laminated pocket cards for aphasia testing. During the pandemic we blew up the pocket cards used for aphasia testing onto 8.5 × 11 sheets of paper that were discarded after each use. Pen lights were encased in sealed plastic bags to facilitate repeated cleanings with gel before and after each patient encounter. Fundoscopic examinations were halted due to the prolonged close interaction. Pupil examinations and cranial nerve examinations are also performed in close proximity to the patient's face, so we required N-95 mask and face shield use for all patient encounters in order to perform these procedures safely.

## Hot and Cold Zones at KCHC

Throughout the hospital patients were cohorted into “hot zones” for COVID-19 positive patients and “cold zones” for COVID-19 negative ones. As much as possible hot and cold zones were separate wards. Patients under investigation for COVID-19 (PUIs) were housed in single rooms in hot zones until their status could be determined for an appropriate ward allocation. However, due to the specialized nature of our Stroke Unit, the Neurology unit became a “mixed zone” unit for much of the surge. Attending rounds started with the COVID-19 negative patients, then PUIs, and finished with COVID-positive patients. Rounds were asynchronous with only the attending and the resident caring for the patient entering the patient's room.

## Stroke Service and a Surprising Increase in Admissions During COVID-19

**Figure 1:**
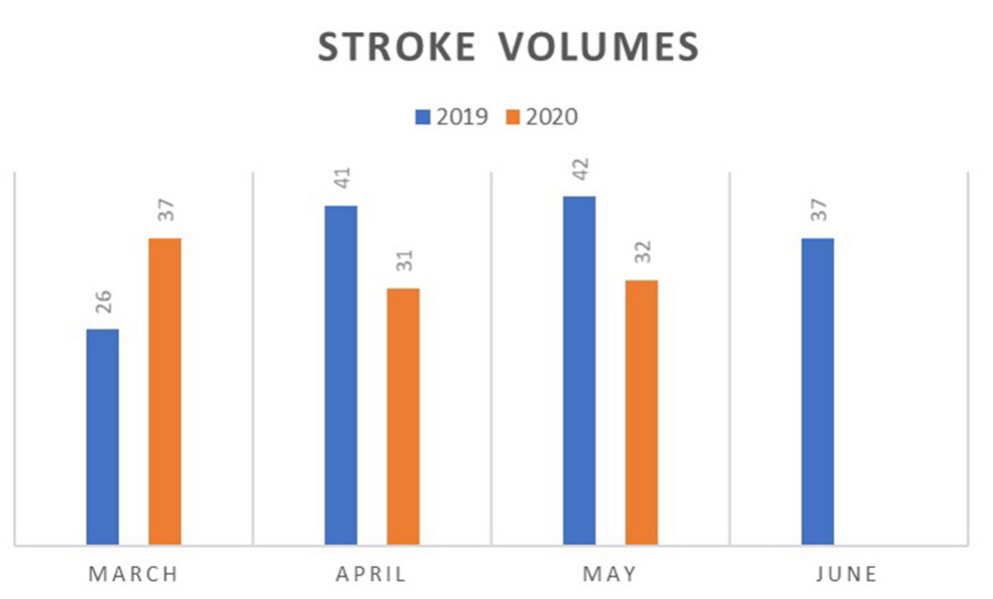
March through June Stroke Volumes (2019 vs 2020).

The Stroke Service developed a stroke cart with interactive video that could be monitored remotely so that when the attending and resident went into the patient's room to examine the patient, the rest of the team could view the interaction and observe social distancing. In particular, this enabled the on-call residents to remotely view the patient's neurologic examination and maintain the quality of care. Unlike other centers, KCHC has seen an increase in both ischemic and hemorrhagic stroke volumes during the month of March of the pandemic in comparison to the same period in 2019 (Figure 1). In the earliest phase of the surge of COVID-19 cases, all the stroke patients were COVID-19 positive. As the pandemic progressed the number of stroke cases decreased and then returned to pre-COVID levels as the patients became COVID negative.

Aside from the new PPE requirements and shortened more distant neurological examinations, stroke codes were minimally affected by COVID-19. We had the same indications for tPA administration and used the same algorithms for thrombectomies. At the surge's peak, when ICU beds were tight and we were able to provide a step-down level of care on our Neurology unit, we modified our tPA administration pathway using the Safety Trial of Low-Intensity Monitoring After Thrombolysis (OPTIMIST) trial as a basis (7). The streamlined OPTIMIST protocol was chosen given that it limits the frequency of patient interactions, while still maintaining patient safety. We administered tPA in the ED and performed the initial 2 h of neuro checks every 15 min as usual. At that point, the patient was transferred to the Neurology unit where neuro checks were performed every hour for 8 h. A non-contrast computerized tomography of the head was performed and, if no hemorrhage was demonstrated, the patient then received neuro checks every 4 h to complete 24 h of post-tPA monitoring. We did not see an increase in complications using this modified tPA administration pathway.

## Point of Care Eeg Overcoming the Challenges During COVID-19

After stroke codes, performing EEG was the next highest risk procedure due to the prolonged interaction time and close proximity to the patient's head. The American Clinical Neurophyisology Society recommendations for minimizing equipment contamination and the amount of time the EEG tech needs to stay in the room were followed (8). They stated that efforts should be made to limit technician exposure to potentially infectious patients; to consider rapid application EEG with disposable, single use caps/ templates; use of antiseptic wipes to clean all surfaces of the equipment that has entered any COVID+/PUI patient room; consider using one use plastic covers to shield EEG equipment in COVID+/PUI rooms; consider keeping the machine outside the patient's room (via long wiring).

Our EEG technicians wore the same PPE as used for stroke codes and we modified the EEG procedures to minimize patient interactions in several ways. We used the novel Bio-Signal group system of disposable electrode strips. We chose this system because it allows for good electrode coverage of most of the brain with 16 electrodes (as opposed to 21 with the traditional 10–20 system) and is rapidly applied (9). The average traditional electrode setup time is 27 min. In addition to deployment time, the tech would normally have to reenter the patient's room to check signal quality at least 3 times for a 24-h study. After training, our EEG technicians were able to apply the novel, rapidly deployable system in 3 min. In addition, we configured the EEG acquisition system to allow the EEG to display on a laptop located outside the patient's room so that the EEG technician could leave the patient's room and still monitor the EEG quality. The studies were uploaded to a server via a sender application on the laptop allowing the neurologists to interpret the studies remotely.

**Figure 2:**
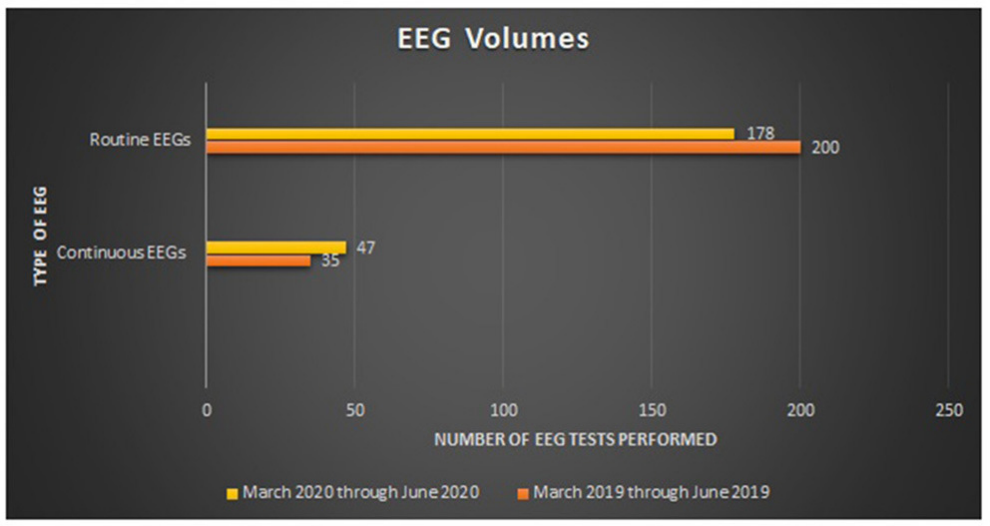
March through June EEG Volumes (2019 vs 2020).

From March 1, 2019 through June 5, 2019, 235 inpatient EEG studies were performed of which 200 were routine and 35 were continuous and for the same time period in 2020, a total of 225 EEG studies were performed (4% decrease), of which 178 were routine (11% decrease) and 47 were continuous (34% increase) (Figure 2). This increase in continuous EEG volumes is in contrast to other institutions which witnessed a dramatic decrease (10).

## Conclusion

The COVID-19 pandemic hit Brooklyn and Kings County Hospital Center hard as it did elsewhere and is continuing to do so today. As a public hospital we made do with limited financial resources and leveraged technology and the resourcefulness of our staff. The Neurology Service attending physicians, residents and technicians altered their daily processes to adapt to the overwhelming nature of this virus. Adhering to strict Infection Control protocols, receiving a crash course in medical management of the COVID-19 patient, modifying our stroke code and examination protocols, and employing advanced technology for the performance of EEG allowed the service to perform its tasks safely and efficiently and to maintain quality of care.

## Author Contributions

HV and SA contributed to the study design, data analysis, and editing. NS and SL provided editorial and have reviewed and approved the final work. All authors contributed to the article and approved the submitted version.

## Conflict of Interest

SA reports support from Bio Signal Group. In addition, SA has a U.S. patent 13/284,886. The remaining authors declare that the research was conducted in the absence of any commercial or financial relationships that could be construed as a potential conflict of interest.
